# IFI27 Integrates Succinate and Fatty Acid Oxidation to Promote Adipocyte Thermogenic Adaption

**DOI:** 10.1002/advs.202301855

**Published:** 2023-08-06

**Authors:** Xuan Cui, Haojie Liu, Ting Shi, Qingwen Zhao, Feiyan Li, Wenjing Lv, Chao Yu, Haiyan Huang, Qi‐Qun Tang, Dongning Pan

**Affiliations:** ^1^ Key Laboratory of Metabolism and Molecular Medicine of the Ministry of Education Department of Biochemistry and Molecular Biology School of Basic Medical Sciences Fudan University Shanghai 200032 China

**Keywords:** brown adipocyte, IFI27, metabolic adaption, mitochondria, thermogenesis

## Abstract

Mitochondria are the pivot organelles to control metabolism and energy homeostasis. The capacity of mitochondrial metabolic adaptions to cold stress is essential for adipocyte thermogenesis. How brown adipocytes keep mitochondrial fitness upon a challenge of cold‐induced oxidative stress has not been well characterized. This manuscript shows that IFI27 plays an important role in cristae morphogenesis, keeping intact succinate dehydrogenase (SDH) function and active fatty acid oxidation to sustain thermogenesis in brown adipocytes. IFI27 protein interaction map identifies SDHB and HADHA as its binding partners. IFI27 physically links SDHB to chaperone TNF receptor associated protein 1 (TRAP1), which shields SDHB from oxidative damage‐triggered degradation. Moreover, IFI27 increases hydroxyacyl‐CoA dehydrogenase trifunctional multienzyme complex subunit alpha (HADHA) catalytic activity in β‐oxidation pathway. The reduced SDH level and fatty acid oxidation in *Ifi27*‐knockout brown fat results in impaired oxygen consumption and defective thermogenesis. Thus, IFI27 is a novel regulator of mitochondrial metabolism and thermogenesis.

## Graphical abstract

Mitochondria are central organelles in energy metabolism which determine the capacity of adipocyte thermogenesis. Mitochondrial matrix protein IFI27 protects SDHB from oxidative damage, and promotes fatty acid oxidation (FAO) by increasing HADHA activity. Loss of IFI27 in adipocytes elicits compromises succinate and fatty acid oxidation in mitochondria and decreases adaptability to cold challenge.

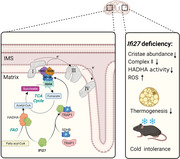

## 1 Introduction

Brown fat tissue (BAT) is a major site of non‐shivering thermogenesis and their mitochondria express uncoupling protein 1 (UCP1) to uncouple respiration from ATP synthesis. Cold or adrenergic stimulation strongly boosts BAT thermogenesis accompanied by enhanced nutrient oxidation and oxygen consumption. During this process mitochondrial respiratory system which consists of four multiheteromeric complexes (Complex I‐IV) transfers electrons liberated by the oxidation of reducing equivalents to molecular oxygen and transports protons from the mitochondrial matrix toward the intermembrane space. The membrane potential and electrochemical gradient of protons across the mitochondrial inner membrane drive proton influx to matrix to generate heat instead of energy.

The thermogenic and metabolic functions of mitochondria in BAT are coupled to changes in mitochondrial proteome and architecture which are adapted specifically to cellular requirements. Proteins involved in fatty acid oxidation, tricarboxylic acid (TCA) cycle and oxidative phosphorylation are enriched in mitochondria of BAT as compared to white adipose tissue (WAT).^[^ ^1^ ^]^ Proteome analysis revealed that chronic cold exposure induced mitochondrial biogenesis, and upregulation of the components of the respiratory chain and TCA cycle even on a “per mitochondria” basis.^[^ ^1^ ^]^ Mitochondrial metabolic adaption is important for adipocyte non‐shivering thermogenesis, and its maladaptation may leads to metabolic diseases associated with mitochondrial dysfunction.^[^ ^2^, ^3^ ^]^

Our previous study identified IFI27 (IFNα inducible protein 27, also known as IFI27L1, ISG12A) as a BAT‐enriched and nuclear‐encoded mitochondrial protein.^[^ ^4^ ^]^ As a member of the interferon‐stimulated gene (ISG) family,^[^ ^5^ ^]^ the expression of *Ifi27* was not induced but was repressed by IFNα treatment in brown adipocytes.^[^ ^4^ ^]^ Activation of IFNα signaling interferes with brown fat thermogenic program and mitochondrial respiration.^[^ ^6^ ^]^ Significantly elevated level of ISGs but reduced electron transport chain abundance was observed in BAT of cold‐exposed UCP1‐KO mice.^[^ ^7^ ^]^ All these data indicated the involvement of ISGs in regulating BAT functions. Indeed, deletion of *Ifi27* in adipocytes caused a broad repression of mitochondrial gene expression.^[^ ^4^ ^]^ However, the requirement of *Ifi27* for BAT physiology in vivo remains elusive. Here we show adipocyte‐specific *Ifi27* knockout mice were intolerant to acute cold challenge, which was associated with aberrant mitochondrial cristae morphology, markedly decreased level of electron transport chain (ETC) Complex II and compromised fatty acid oxidation in BAT. Mechanistically, IFI27 interacts with SDHB (succinate dehydrogenase complex iron sulfur subunit B) and HADHA (hydroxyacyl‐CoA dehydrogenase trifunctional multienzyme complex subunit alpha), acting as a dual regulator of SDHB stability and HADHA activity in brown adipocytes. These findings indicate IFI27 is required for maintaining mitochondrial metabolic plasticity, especially in response to thermogenic stimuli in brown adipocytes.

## 2 Results

### 2.1 IFI27 is a Mitochondrial Matrix Protein

**Figure 1:**
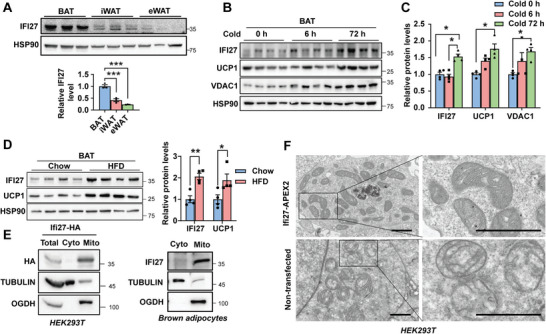
IFI27 localizes to BAT mitochondrial matrix. A) Western blot analysis of IFI27 protein and its quantification (bottom) in brown and white adipose tissues of C57BL/6 mice. *n* = 3 each group. B) IFI27 level in BAT after cold challenge for indicated time. *n* = 4 each group. C) Quantification of protein levels in (B). *n* = 4 each group. D) Western blot analysis of indicated protein and their quantification in BATs of mice fed on a high fat diet (HFD) for 11 weeks. *n* = 4 each group. E) Western blot analysis of IFI27 in HEK293T (left) and brown adipocytes (right). A plasmid expressing *Ifi27‐HA* was transfected into HEK293T cells. Immortalized brown preadipocytes were differentiated into mature adipocytes. Mitochondria were separated from the cytosol fraction and subject to western blot with indicated antibodies. The experiment was repeated for three times. F) Transmission electron microscope (TEM) analysis of the localization of IFI27‐APEX2. The region inside the black box was enlarged for clearer view. Scale bar: 1 µm. For statistical analyses, one‐way ANOVA analysis of variance and Tukey's post hoc tests were performed in (A), two‐way ANOVA analysis of variance and Tukey's post hoc tests were performed in (C), and two‐tailed unpaired Student's *t*‐test was performed in (D). The data shown are mean ± SEM. *p < 0.05; **p < 0.01; *p < 0.001.

*Ifi27* was first identified as a BAT‐enriched gene relative to epididymal WAT (eWAT) in our published RNA‐seq data (GEO: GSE56367).^[^ ^8^ ^]^ Its mRNA expression profile in mouse tissues has been explored by quantitative PCR,^[^ ^4^ ^]^ but not been verified at the protein level since there was no any commercial IFI27 antibody available. We developed a rabbit polyclonal antibody against synthetic peptide containing 105 to 122 amino acids of IFI27. The antibody specificity was validated in *Ifi27‐*knockdown brown adipocytes (Figure S1A, Supporting Information). Then immunoblotting analysis with this antibody demonstrated the level of IFI27 was high in interscapular BAT, whereas inguinal WAT (iWAT) and eWAT expressed a relatively low amount (**Figure** **1**A). IFI27 also existed substantially in heart and skeletal muscle, consisting with their high mitochondrial contents as indicated by the outer mitochondrial membrane protein VDAC1 (voltage dependent anion channel 1) expression (Figure S1B, Supporting Information). Cold exposure for 3 days at 4 °C or high fat diet‐feeding increased IFI27 expression in BAT, which was positively correlated with the UCP1 protein level (Figure 1B–D). The elevated IFI27 level was observed in browning iWAT induced by cold or β3‐adrenergic agonist treatment as well (Figure S1C,D, Supporting Information).

IFI27 was shown to localize to mitochondria in brown adipocytes by immunofluorescence staining.^[^ ^4^ ^]^ Differential centrifugation followed by western blot analysis reinforced that both ectopically expressed IFI27‐HA in HEK293T cells and endogenous IFI27 in brown adipocytes were confined to mitochondria exclusively (Figure 1E). Mitochondria consist of four compartments: outer membrane (OM), inner membrane (IM), intermembrane space (IMS) and matrix. To pinpoint the subcompartmental localization inside mitochondria, we transfected the *Ifi27*‐APEX2 plasmid to HEK293T cells and performed electron microscopy (EM) imaging.^[^ ^9^ ^]^ The non‐transfected cells had palely stained matrix. In contrast, the dark region associated with IFI27‐APEX2 was mainly in the mitochondrial matrix but not in the IMS (Figure 1F), indicating IFI27 was mainly in the mitochondrial matrix.

### 2.2 Loss of IFI27 Causes a Remarkable Decrease in Mitochondrial Cristae

**Figure 2:**
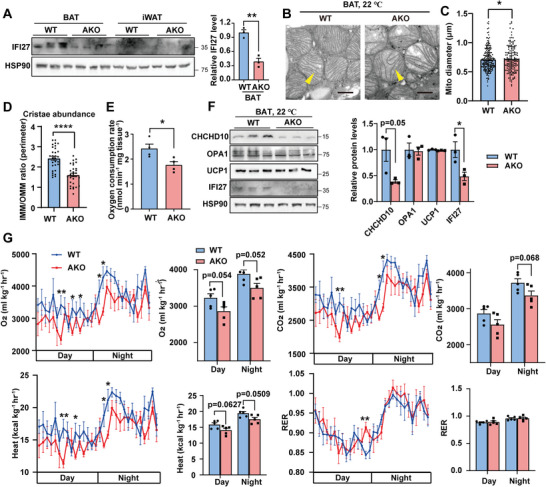
AKO mice possess remarkably decreased mitochondrial cristae. A) Western blot analysis of the IFI27 protein and its quantification in fat tissues of the WT and AKO mice. *n* = 3 each genotype. B) TEM illustrated mitochondrial structure in BAT of the WT and AKO mice. Yellow arrowheads indicate mitochondrial cristae. Scale bar: 500 nm. C,D) Mitochondrial diameter (C) and cristae abundance (D) in BAT of the WT and AKO mice. Diameter was determined by measuring 100 mitochondria in TEM images. Cristae abundance was shown as the perimeter ratio of inner to outer mitochondrial membranes (*n* = 30 each group). E) BAT oxygen consumption rate was measured by Clark‐type oxygen electrodes. *n* = 4 each genotype. F) Western blot analysis of the indicated proteins and their quantification in BAT of WT and AKO mice. *n* = 3 each genotype. G) Metabolic cage analyses for WT and AKO mice. Oxygen consumption, carbon dioxide production, heat production and respiratory exchange ratio (RER) were measured. *n* = 5 each genotype, 8‐week‐old male mice. For statistical analyses, two‐tailed unpaired Student's *t*‐test was performed. The data shown are mean ± SEM. *p < 0.05; **p < 0.01; ****p < 0.0001.

To investigate the physiological role of IFI27 in adipose tissues, we generated mice lacking *Ifi27* in adipose tissues (AKO) by crossing *Ifi27* floxed mice to *Adipoq*‐*Cre* lines (Figure S2A, Supporting Information). *Ifi27‐*knockout mice lost nearly 70% of IFI27 protein in BAT without disturbance in heart and skeletal muscle IFI27 expression (**Figure** **2**A and Figure S2B, Supporting Information). *Ifi27*‐AKO mice were indistinguishable from WT littermates in appearance when they were raised at ambient temperature (22 °C) and fed a chow diet. They consumed comparable amount of food and had similar level of blood glucose and body weight (Figure S2C–E, Supporting Information). Hematoxylin and Eosin (H&E) staining showed normal histological morphology of BAT and iWAT in AKO mice as compared to WT littermates (Figure S2F, Supporting Information).

Given IFI27 is a mitochondrial matrix protein, we were especially intrigued by whether there were any alterations in mitochondrial function after depleting *Ifi27*. Remarkably, transmission electron microscopy (TEM) revealed slightly swelling mitochondria and a considerable decrease in the number of cristae in *Ifi27*‐AKO BAT (Figure 2B–D). Correspondingly, the isolated BATs from AKO mice had a significant lower oxygen consumption rate (OCR) and the CHCHD10 (coiled‐coil‐helix‐coiled‐coil‐helix domain containing 10) level, which is a component of mitochondrial contact site and cristae‐organizing system (MICOS) complexes (Figure 2E,F). OPA1 mitochindrial dynamin like GTPase is an important regulating factor in the establishment and maintenance of cristae architecture.^[^ ^10^ ^]^ However, the levels of OPA1 and the inner member protein UCP1 were not affected by loss of *Ifi27* (Figure 2F). Then, we measured the metabolic parameters and energy expenditure using indirect calorimetry in WT and AKO mice at ambient temperature. *Ifi27* knockout mice tended to have a decreased systemic oxygen consumption (*p* = 0.05 at both day and night cycles), decreased carbon dioxide release (*p* = 0.068 at night cycle) and heat production (*p* = 0.06 during daytime and *p* = 0.05 at night cycle) as compared with WT mice, but keep a similar respiratory exchange ratio (RER) (Figure 2G).

Mitochondrial DNA exists in the mitochondrial matrix, which encodes 13 subunits of respiration complex, 2 ribosomal RNAs and 22 transfer RNAs. We examined the expression level of mtDNA‐encoded transcripts, and there was no difference between the WT and AKO mice (Figure S2G, Supporting Information).

### 2.3 Adipocyte‐Specific *Ifi27* Knockout Mice are Cold Intolerant

**Figure 3:**
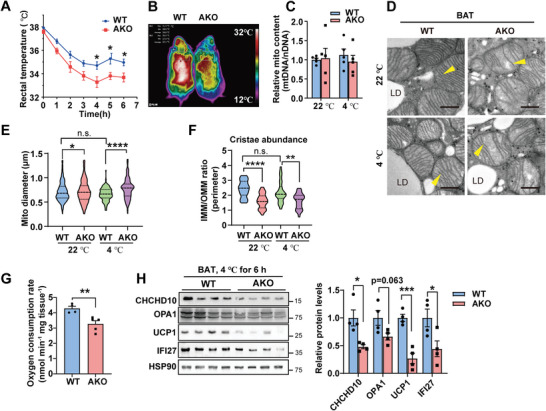
*Ifi27* knockout mice are cold intolerant. A) Rectal temperature of the WT and AKO mice at 4 °C without food supply. *n* = 5 each genotype, 8‐week‐old male mice. B) Mice were subjected to 4 °C for 6 h without food supply. Dorsal skin temperature was measured by an infrared camera. C) BAT mitochondrial content was determined by measuring mitochondrial DNA level. Mice were kept at 22 or 4 °C for 6 h. *n* = 4–5 each genotype. D) TEM analysis of BAT mitochondrial structure for mice at 22 or 4 °C for 6 h. Yellow arrowheads indicated mitochondrial cristae. LD: lipid droplet. Scale bar: 500 nm. E,F) Quantification of mitochondrial diameter (E) and the cristae abundance (F) in TEM images. *n* = 100 mitochondria each group in (E), and *n* = 30 each group in (F). n.s: not significant. G,H) Mice were exposed to 4 °C for 6 h. BAT oxygen consumption rate (G) and western blot analysis of the indicated proteins and their quantification (H) were shown. *n* = 4–5 each genotype. For statistical analyses, two‐way ANOVA analysis of variance and Bonferroni's post hoc tests were performed in (A), and unpaired two‐tailed Student's *t‐*tests were performed in (E–H). The data shown are mean ± SEM. *p < 0.05; **p < 0.01; ****p < 0.0001.

The intact mitochondrial structure and function in BAT is essential for the non‐shivering thermogenesis upon cold challenge.^[^ ^11^, ^12^ ^]^ We queried whether *Ifi27*‐AKO mice could keep intact thermogenic capability to combat cold in the case of unusual mitochondrial morphology. The average rectal temperature of *Ifi27*‐AKO mice dropped from 37.6 to 33.7 °C within 6‐h 4 °C exposure without food supply, while the WT mice maintained body temperature at 35 °C (**Figure** **3**A). Infrared thermography also demonstrated a significant decrease in dorsal surface temperature in AKO mice after 6‐h cold challenge (Figure 3B). The mitochondrial content was similar between genotypes. Acute cold exposure did not further aggravate mitochondrial swelling or reduction in cristae in AKO BATs as reflected by quantification of mitochondrial diameters and cristae abundance (Figure 3C–F). The BAT isolated from cold‐exposed AKO mice consumed 23.6% less oxygen than that of the WT mice (Figure 3G). The level of UCP1 was dramatically decreased in the AKO mice after the severe cold challenge (Figure 3H).

WAT browning is an alternative thermogenic mechanism and can be induced by long‐term cold exposure. When the mice were exposed to 4 °C for 3 days with free access to food and water, the AKO mice still had lower rectal body temperature (Figure S3A, Supporting Information). 3‐day cold acclimation was sufficient to induce browning of iWAT in WT mice, manifested by the appearance of plenty of adipocytes containing multilocular lipid droplets in iWAT (Figure S3B, Supporting Information). *Ifi27*‐AKO mice had much fewer beige adipocytes, reduced UCP1 immunohistochemistry staining, less mitochondrial mass, and swelling mitochondria in iWATs (Figure S3B–E, Supporting Information). The cristae abundance and oxygen consumption rate showed no difference between genotypes (Figure S3F,G, Supporting Information). Western blot revealed the level of UCP1 were lower in iWAT of AKO mice (Figure S3H, Supporting Information). The expression of genes involved in calcium and creatine cycles, and mitochondrial DNA‐encoding transcripts were similar (Figure S3I,J, Supporting Information). The data here demonstrate that deleting *Ifi27* in fat tissues blunts adipocytes thermogenic revival during cold adaption which results in the AKO mice cold intolerant.

### 2.4 IFI27 Ablation Elicits Succinate Accumulation in BAT Due to a Decrease in SDHB Level

**Figure 4:**
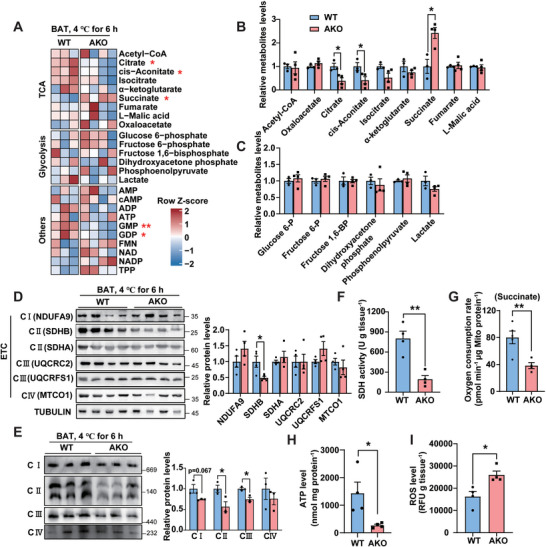
IFI27 ablation elicits succinate accumulation in BAT due to a decrease in SDHB level. A) Heat map of metabolomics analysis in BAT. Mice were challenged at 4 °C for 6 h. *n* = 3–4 each genotype, 12‐week‐old female mice. B) The relative level of TCA metabolites in BAT measured by LC‐MS/MS assay. *n* = 3–4 each genotype. C) The relative level of glycolysis‐related metabolites in BAT measured by LC‐MS/MS assay. *n* = 3–4 each genotype. D) Western blot analysis of ETC‐related proteins and their quantification in BAT of mice after 6 h cold exposure. *n* = 4 each genotype. E) Mice were treated as in (A). BAT mitochondria were isolated and underwent blue native‐PAGE analysis. Right panel showed the quantification of respiratory complexes. *n* = 3 each genotype. F–I) Mice were treated as in (A). SDH activity (F), mitochondrial oxygen consumption rate with succinate as substrates (G), ATP (H) and ROS (I) levels were determined in BAT. *n* = 4–5 each genotype. For statistical analyses, unpaired two‐tailed Student's *t*‐tests were performed. The data shown are mean ± SEM. *p < 0.05; **p < 0.01.

Mitochondria are energy metabolism hub. To discern whether there is metabolic defects existing in BAT of the AKO mice, we employed targeted metabolites analysis which covers a spectral array of endogenous metabolites involved in energy generation in the fed mice acutely exposed to a cold environment (**Figure** **4**A). The only prominently increased TCA metabolite was succinate, accompanied by slightly decreased levels of citric acid and cis‐aconitic acid in AKO mice BAT (Figure 4B). In contrast, there was no difference in the levels of glycolysis metabolites (Figure 4C). Correspondingly, the WT and AKO mice showed comparable levels of glycolysis key enzymes in BAT (Figure S4A, Supporting Information).

The accumulation of succinate strongly indicated a decrease in succinate dehydrogenase (SDH) activity. In addition to its responsibility in TCA cycle, SDH serves as the complex II of oxidative respiratory chains.^[^ ^13^ ^]^ The expression of the key enzymes of TCA, including CS (citrate synthase), IDH3A (isocitrate dehydrogenase 3 catalytic subunit alpha) and OGDH (oxoglutarate dehydrogenase), was not affected by deletion of *Ifi27* (Figure S4B, Supporting Information). However, when we checked the levels of the representative subunit of respiratory chain complexes, a reduced SDHB expression was disclosed (Figure 4D). SDHB associated with SDHA in matrix is anchored off SDHC‐SDHD subunits that are embedded in the inner mitochondrial membrane, forming complex II.^[^ ^14^ ^]^ It is reported macrophages stimulated with lipopolysaccharides (LPS) selectively depleted SDHB, but not SDHA, leading to a decrease in the assembled Complex II and SDH enzymatic activity.^[^ ^15^ ^]^ Our blue native‐PAGE revealed a dramatic decrease in fully assembled complex II (220KDa), and the assembly intermediate composed of SDHA and SDHB (about 110KDa)^[^ ^16^ ^]^ (Figure 4E). The level of assembled Complex III was also shown a slight reduction (Figure 4E). As expected, the SDH activity and the mitochondrial oxygen consumption rate with succinate as substrates were significantly lower in BAT of cold‐challenged AKO mice (Figure 4F,G). Oxidative respiratory complex is essential for ATP production and is a major site for generation of reactive oxygen species (ROS). The BAT of *Ifi27*‐AKO mice harboring declined assembled respiratory complexes produced less ATP but more ROS (Figure 4H,I).

We next evaluated the effects caused by ablation of *Ifi27* in cultured adipocytes. Knockdown of *Ifi27* by small hairpin RNAs (shRNAs) in brown adipocytes significantly reduced the SDHA and SDHB levels, decreased mitochondrial membrane potential and oxygen consumption rate, but spurred ROS production (Figure S4C–F, Supporting Information). Here, we saw a decreased SDHA level in the *Ifi27‐*knockdown adipocytes (Figure S4C, Supporting Information), but it did not happen in the AKO BATs (Figure 4D). When silencing *Sdhb* in brown adipocytes by shRNAs, the SDHA level decreased by 53% (Figure S4G, Supporting Information). Previous studies observed reduced SDHA levels in the SDHB‐deficient fibroblasts,^[^ ^17^ ^]^ supporting a proposal that whatever *Sdh* gene inactivation may trigger a destabilization and proteolysis of SDH subunits.^[^ ^18^ ^]^ The unaffected SDHA in the AKO BATs might be associated with a relatively minor decline of SDHB in vivo as compared to in both IFI27 and SDHB knockdown cells (the SDHB level was decreased to 50%, 24% and 22% in the AKO BAT, IFI27‐ and SDHB‐knockdown cells, respectively). Correlating well with the lower level of SDHA/B in *Ifi27*‐knockdown cells, the depressed SDH activity and accumulated intracellular and extracellular succinate were observed (Figure S4H–J, Supporting Information). These data suggest that cell‐autonomous mechanisms are involved in modulating the SDH level upon *Ifi27* deletion.

### 2.5 IFI27 Facilitates SDHB Interacting With the Mitochondrial Chaperone TRAP1

**Figure 5:**
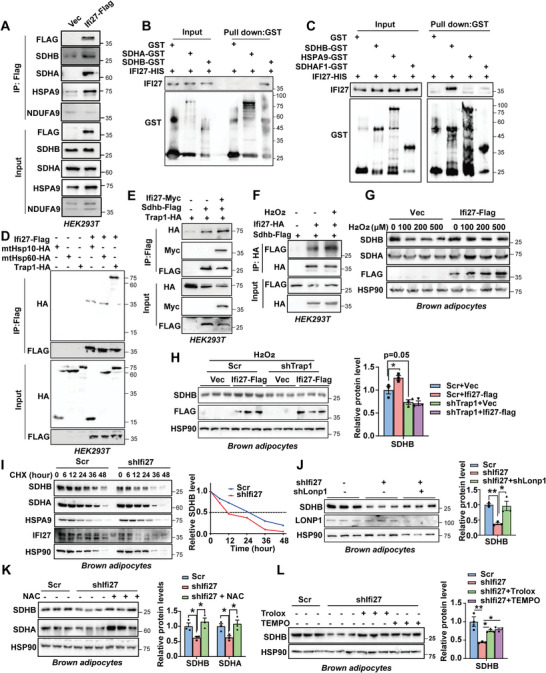
IFI27 serves as an adaptor to link SDHB to TRAP1. A) Immunoprecipitation and western blot analysis of the indicated proteins in the HEK293T cells transfected with *Ifi27*‐*Flag*. B,C) GST pull‐down assays followed by western blot showed IFI27 interacted with SDHB directly. D,E) Plasmids as indicated were transfected into HEK293T cells. Immunoprecipitation was performed 48 h after transfection followed by western blot analysis. The experiment was repeated for three times. F) SDHB and IFI27 were ectopically expressed in HEK293T cells by plasmid transfection. Hydrogen peroxide (100 µm) treated cells for 6 h followed by immunoprecipitation with HA antibody. The experiment was repeated for three times. G) Immortalized brown preadipocytes expressing *Ifi27‐Flag* differentiated into mature adipocytes. Hydrogen peroxide treated adipocytes for 12 h followed by western blot analysis. H) Lentivirus expressing IFI27‐Flag or vector transduced *Trap1* knockdown adipocytes on differentiation day 2 and day 4. Hydrogen peroxide (100 µm) treated cells for 12 h followed by western blot analysis on day 6. The right panel showed the SDHB protein quantification. *n* = 3 each group. I) Lentiviral shRNA against *Ifi27* transduced preadipocytes followed by induction to mature adipocytes. Cycloheximide (CHX) treated the cells for indicated time and western blot analyzed the protein levels (left). Right, quantification of the SDHB level over time. The experiment was repeated for three times. J) *Ifi27* knockdown preadipocytes differentiated to day 3. Then *Lonp1* was knocked down by transducing lentivirus shRNAs. The SDHB and LONP1 levels were detected by western blot on day 6. Right, quantification of the SDHB level. *n* = 3 each group. K,L) Western blot analysis and the quantification of indicated proteins in *Ifi27‐*knockdown brown adipocytes. N‐acetyl‐L‐cysteine (NAC 2 mm) (K), Trolox (100 µm) or TEMPO (25 µm) (L) treated the cells during differentiation. *n* = 3 each group. For statistical analyses, one‐way ANOVA analysis of variance and Tukey's post hoc tests were performed in (H, J–L). The data shown are mean ± SEM. *p < 0.05; **p < 0.01.

Since the respiratory complex II underwent the most dramatic change upon IFI27 ablation, we then sought to discern how deficiency of *Ifi27* elicited declined SDH expression. In view of the fact that there was no alteration in the *Sdhb* mRNA level in BAT of the cold‐exposed AKO mice (Figure S5A, Supporting Information), we predicted that IFI27 affects SDHB level post‐transcriptionally. SDHB is encoded by nuclear DNA, and the newly synthesized pro‐SDHB protein is translocated from cytoplasm to mitochondrial matrix where it undergoes folding, iron‐sulfur (Fe‐S) insertion and assembling with other subunits of SDH. HSPA9 (Heat shock protein family A member 9, also known as HSP70) together with the scaffold ISCU (Iron‐sulfur cluster assembly enzyme) and the co‐chaperon HSCB (HscB mitochondrial iron‐sulfur cluster chaperone) constitutes the Fe‐S transfer complex incorporating three Fe‐S clusters into SDHB.^[^ ^16^ ^]^ To test whether IFI27 is involved in the SDHB maturation in mitochondrial matrix, an immunoprecipitation assay between IFI27 and SDHA/B or the Fe‐S transfer complex was performed. Ectopically expressed IFI27‐HA did not interact with SDHA, ISCU or HSCB, but bound to SDHB and HSPA9 in HEK293T cells (Figure S5B,C, Supporting Information). When we overexpressed IFI27‐FLAG in HEK293T cells by a plasmid transfection and precipitated IFI27 with Flag antibody, endogenous SDHB, SDHA and HSPA9 were detected in the IFI27‐Flag immunoprecipitates, suggesting they are in the same complex (**Figure** **5**A). As a control, the NDUFA9 subunit of complex I was not present in the complex (Figure 5A). The physical interaction of IFI27 with SDHB, SDHA and HSPA9 was further verified by the GST pull‐down assay with purified recombinant proteins in vitro. The data demonstrated that IFI27‐His recombinant protein only bound to SDHB directly, leaving out SDHA and HSPA9 (Figure 5B,C). The LYR factor SDHAF1 (SDH complex assembly factor 1), which mediates transfer of Fe‐S clusters to SDHB,^[^ ^18^, ^19^ ^]^ did not associate with IFI27 either (Figure 5C).

Mitochondrial molecular chaperones interact with nascent polypeptides facilitating their folding or aiding misfolded or damaged proteins to be repaired. It is reported that SDHB is one of the clients for mitochondrial chaperone HSP90 paralog TRAP1 (TNF receptor associated protein 1).^[^ ^20^ ^]^ We verified that loss of *Trap1* in brown adipocytes led to a significant decrease in both SDHA and SDHB levels, without perturbation of IFI27 expression (Figure S5D, Supporting Information). Intriguingly, IFI27 interacted with TRAP1, but not other mitochondrial chaperones like HSP70/HSPA9 (Figure 5C), HSP10/HSPE1 or HSP60/HSPD1 (Figure 5D). Moreover, the association between TRAP1 and SDHB was enhanced by the existence of IFI27 (Figure 5E).

### 2.6 IFI27 Stabilizes SDHB in Face of Mitochondrial Stress

Cold exposure increases ROS formation in brown adipocytes.^[^ ^21^ ^]^ SDHB is supposed to be fragile to oxidative stress since Fe‐S clusters are particularly sensitive to ROS.^[^ ^22^ ^]^ Cold exposure for 1 or 3 days did not induce *Trap1* expression on its mRNA level (Figure S5E, Supporting Information). The upregulated *Ifi27* (shown in Figure 1B) might be to accomplish the heavy demand for stabilizing SDHB protein upon cold stress. Hydrogen peroxide (H_2_O_2_) treatment which was used to mimic the oxidative stress decreased the SDHB level at a concentration of 100 µm, and did not affect IFI27 protein levels (Figure S5F, Supporting Information). Instead, oxidative stress enhanced the interaction between IFI27 and SDHB (Figure 5F). Overexpressed IFI27 protected SDHB from H_2_O_2_‐induced reduction in its protein level in both brown fat cells and HEK293T cells (Figure 5G and Figure S5G, Supporting Information). However, IFI27 could not increase SDHB level any longer when TRAP1 was knocked down, suggesting TRAP1 is required for IFI27‐mediating SDHB protection (Figure 5H). By contrast, IFI27 did not guard SDHA against an oxidative damage‐associated decrease in the protein level (Figure S5G, Supporting Information). The level of UQCRFS1 (ubiquinol‐cytochrome c reductase, Rieske iron‐sulfur polypeptide 1), which is the only subunit with Fe‐S clusters in complex III, was not affected by loss of *Ifi27* in BAT either (shown in Figure 4D), indicating SDHB is a relative specific client for IFI27. The absence of IFI27 did not cause mitochondrial unfolded protein reaction (UPR) as the expression of mitochondrial UPR marker genes kept comparable to the WT mice (Figure S5H, Supporting Information).

To determine how IFI27 regulates SDHB protein level, we checked the half‐life time of SDHB with the presence or absence of IFI27. Cycloheximide chase assay revealed expressing IFI27 stabilized SDHB in HEK293T cells (Figure S5I, Supporting Information). On the contrary, once *Ifi27* was knocked down in brown adipocytes, the half‐life time of endogenous SDHB decreased from 24 to 12 h, whereas the turnover time of SDHA and HSPA9 was not changed (Figure 5I). The ATP‐dependent mitochondrial lon peptidase 1 (LONP1) is the main protease to degrade damaged and oxidized proteins in the matrix compartment and is reported to degrade SDHB.^[^ ^18^ ^]^ Indeed, repression of *Lonp1* expression partly rescued the decline of SDHB in *Ifi27*‐knockdown brown fat cells (Figure 5J). In light of the pivotal role of ROS in pathology of *Ifi27* ablation, we tried to use a well‐known antioxidant N‐acetyl cysteine (NAC) to rescue the decreased SDHB in knockdown cells. NAC treatment did not alter SDHB and SDHA protein levels in the control brown adipocytes (Figure S5J, Supporting Information). However, NAC could fully recover the decrease in SDHB and SDHA caused by *Ifi27* silencing (Figure 5K). Another two ROS scavengers Trolox and TEMPO^[^ ^23^ ^]^ showed the similar effects as NAC (Figure 5L).

### 2.7 IFI27 Interacts with HADHA to Modulate its Activity

**Figure 6:**
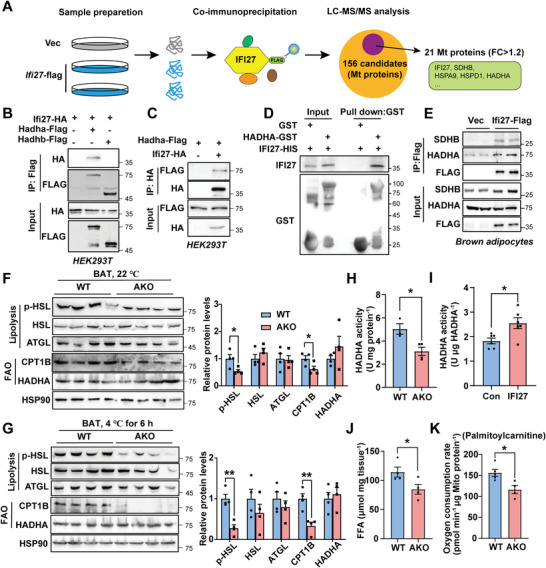
IFI27 binding to HADHA enhances HADHA catalytic activities. A) Schematic of the IFI27 interaction discovery. B,C) Plasmids as indicated were transfected into HEK293T cells. Immunoprecipitation was performed 48 h after transfection followed by western blot analysis. D) GST pull‐down assays following by western blot showed IFI27 interacted with HADHA directly. E) *Ifi27‐Flag* lentivirus transduced brown preadipocytes followed by differentiation into adipocytes. Flag antibody was used for immunoprecipitation and western blot with indicated antibodies was performed. F,G) Western blot analysis detected levels of the indicated proteins in BAT of the WT and AKO mice at 22 (F) and 4 °C for 6h (G). The right panels showed the protein quantification. *n* = 4 each genotype. H) The dehydrogenase activity of HADHA in BAT of the WT and AKO mice at 4 °C. I) Purified GST‐HADHA dehydrogenase activity was measured in vitro in the presence or absence of IFI27 protein. J) Quantification of free fatty acids in BAT of the WT and AKO mice after 4 °C exposure for 6 h. *n* = 4 each genotype. K) Oxygen consumption rate of BAT mitochondria with palmitoylcarnitine and malate as substrates. The mitochondria were isolated from cold‐exposed mice BAT. *n* = 4–5 each genotype. For statistical analyses, unpaired two‐tailed Student's *t*‐tests were performed. The data shown are mean ± SEM. *p < 0.05; **p < 0.01.

To fully perceive the mechanisms involved in cold intolerance of the AKO mice, we performed immunoprecipitation in combination with mass spectrometry (MS) analysis in brown adipocytes to distinguish the IFI27‐interacting proteins unbiasedly (**Figure** **6**A). Based on MitoCarta3.0,^[^ ^24^ ^]^ total 156 mitochondrial proteins were identified, of which 21 proteins displayed 1.2‐fold enrichment in association with IFI27‐Flag beads compared with the IgG control (Table S1, Supporting Information). Not surprisingly, SDHB was within the IFI27 protein interaction networks. HSPA9 (mtHSP70), HADHA, and HSPD1 (mtHSP60) were the top 3 proteins with the highest percent of protein coverage. No interaction of HSPA9 or HSPD1 with IFI27 has been verified in Figure 5C,D. HADHA is one of the subunits of hydroxyacyl‐CoA dehydrogenase trifunctional multienzyme complex, which catalyzes the last three reactions of the mitochondrial fatty acid β‐oxidation. HADHA carries the 2,3‐enoyl‐coA hydratase and the 3‐hydroxyacyl‐coA dehydrogenase activities in β‐oxidation pathway. IFI27 interacted with HADHA, but not HADHB, when each protein was ectopically expressed in HEK293T cells (Figure 6B,C). Purified GST‐HADHA protein directly associated with IFI27 in vitro (Figure 6D). More importantly, we can detect IFI27‐Flag interacted with endogenous SDHB and HADHA in brown adipocytes (Figure 6E).

Brown adipocytes consume large amounts of triglycerides as energy substrates. Following the direction of IFI27 teaming up with HADHA, we wonder whether IFI27 ablation influences lipid metabolism. Although the level of HADHA was not affected by deletion of *Ifi27*, its activity in AKO BAT was decreased by 38.2% (Figure 6F–H). In vitro enzymatic activity assay confirmed the appearance of IFI27 augmented HADHA catalytic activity (Figure 6I). Furthermore, decreased levels of phosphorylated HSL (hormone‐sensitive lipase) and CPT1B (carnitine palmitoyltransferase 1B) were observed in AKO BAT at both 22 and 4 °C (Figure 6F,G). In line with this, loss of IFI27 resulted in less free fatty acid contents in the AKO BAT after 4 °C exposure and compromised mitochondrial oxygen consumption rate when palmitoylcarnitine and malate were provided as respiratory substrates (Figure 6J,K), confirming that the AKO mice possessed an impaired capability at lipid mobilization and utilization.

### 2.8 Loss of IFI27 Alters the Composition of Triglyceride and Glycerophospholipid

**Figure 7:**
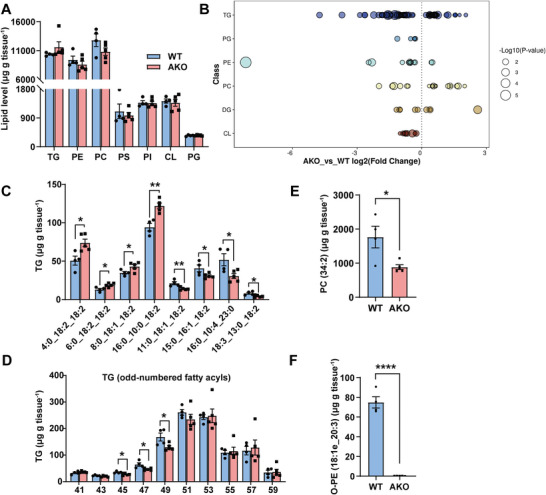
The alteration in molecular species of lipids in the AKO mice BAT. A) Lipidomic analysis of BAT isolated from WT and AKO mice raised at 22 °C. The overall amount of triglycerides and phospholipids was quantified as µg g^−1^ tissue. WT *n* = 4, AKO *n* = 5. TG: triglyceride; PE: phosphatidylethanolamine; PC: phosphatidylcholine; PS: phosphatidylserine; PI: phosphatidylinositol; CL: cardiolipin; PG: phosphatidylglycerol. B) Log2 fold changes in lipid species (AKO versus WT mice, *p* < 0.05). DG: diglyceride. C) The content of the indicated triglyceride species in BAT of WT (*n* = 4) and AKO (*n* = 5) mice. D) The content of the major triglyceride species with odd‐numbered acyl chains. WT *n* = 4, AKO *n* = 5. E) The content of the individual PC (34:2) in BAT of WT (*n* = 4) and AKO (*n* = 5) mice. F) The content of the individual ether PE (18:1e_20:3) in BAT of WT (*n* = 4) and AKO (*n* = 5) mice. For statistical analyses, unpaired two‐tailed Student's *t*‐tests were performed. The data shown are mean ± SEM. *p < 0.05; **p < 0.01; ****p < 0.0001.

HADHA is reported to remodel cardiolipin (CL) in human cardiomyocytes and its mutation disrupts cristae structure.^[^ ^25^ ^]^ We were particularly interested in the cardiolipin composition since the HADHA activity appeared low in the AKO BAT. Thus mass‐spectrometry‐based lipidomic analysis was performed to characterize the composition of both triglyceride and glycerophospholipid species in the BAT of AKO mice at room temperature. The overall abundance of triglyceride (TG) and subspecies of glycerophospholipids was comparable between the WT and AKO groups (**Figure** **7**A). However, there were numerous significant changes (*p* < 0.05) in the composition of each species (Figure 7B and Table S2, Supporting Information). Using a significance level of Variable Importance in Projection (VIP) score >1 and *p* < 0.05, total 8 kinds of TG were dramatically altered in abundance. Interestingly, the 4 increased TGs contained even‐chain fatty acids, however, the 4 decreased TGs contained odd‐chain fatty acids (Figure 7C). When all the TGs were sorted by the carbon number of fatty acyl chains, the AKO mice carried less 45C‐, 47C‐, and 49C‐TGs in the BAT (Figure 7D). Cold exposure increases TG species containing odd‐numbered fatty acyl chains,^[^ ^26^ ^]^ which likely reflects the activation of BAT thermogenesis. Thus IFI27 deficiency gave rise to a TG profile associated with a depressed metabolic and thermogenic activity in BAT.

Phospholipids are major structural components of cell membranes. Phosphatidylcholine PC (34:2) is the most abundant individual PC species in BAT (Table S2, Supporting Information). AKO mice had 50% less PC (34:2) (VIP > 16) and 24% less 34C‐PC in BAT (Figure 7E and Figure S6A, Supporting Information). Alkyl‐ether phosphatidylethanolamine (O‐PE) contains a glycerol backbone with a fatty alcohol attached at sn‐1 position by an ether bond, which is synthesized in the peroxisome and endoplasmic reticulum (ER). Mitochondria contain abundant peroxisome‐derived ether PEs, and ether lipids regulate adipocyte thermogenesis via affecting mitochondrial functions.^[^ ^27^ ^]^ Knocking down expression of GNPAT (glyceronephosphate o‐acyltransferase), which is responsible for the first step in ether phospholipid synthesis, markedly decreased the amount of ether lipids in mitochondrial and inhibited brown adipocyte oxygen consumption.^[^ ^27^ ^]^ Strikingly, O‐PE (18:1e_20:3) almost vanished from the AKO mice BAT (WT 74.87 µg g^−1^ tissue vs AKO 0.26 µg g^−1^ tissue, VIP > 5) (Figure 7F). The total abundance of CL and its precursor phosphatidylglycerol (PG) was not affected by IFI27 depletion (Figure 7A), except that 9 minor CLs and 5 subspecies of PGs were slightly decreased in AKO mice (*p* < 0.05, VIP < 1) (Figure S6B,C, Supporting Information). Significantly lower level of PG with 36C‐acyl chains, which is one of the major PG species, was also observed in the AKO BAT (Figure S6D, Supporting Information). On the basis of these observations, we concludes that deficiency of IFI27 interferes with both TG and phospholipid metabolism.

## 3 Discussion

Mitochondria engage pleiotropic mechanisms to maintain cell fitness and energy homeostasis in the changing environment.^[^ ^28^ ^]^ We discover that IFI27 is a novel regulator on mitochondrial metabolism upon cold challenge. As a mitochondrial matrix protein, IFI27 in adipose tissues was induced by 3‐day cold exposure and high fat diet (HFD) feeding (Figure 1B,D and Figure S1C, Supporting Information). Hydrogen peroxide treatment did not affect the IFI27 level in brown adipocytes (Figure S5F, Supporting Information), indicating the molecular mechanism for cold‐induced IFI27 was not likely a feedback to ROS accumulation. Based on the previous data that deleting the transcription factor peroxisome proliferator‐activated receptor delta (PPARδ) significantly upregulated IFI27,^[^ ^4^ ^]^ we predict the PPARδ transcriptional complex responds to cold and HFD to regulate the expression of IFI27. Non‐liganded PPARδ is a potent transcription inhibitor by binding with corepressors NCOR2 (nuclear receptor corepressor 2) and class I histone deacetylases.^[^ ^29^ ^]^ PPARδ is stimulated by non‐esterified fatty acids from lipolysis upon cold stimuli^[^ ^30^ ^]^ or fatty acid‐rich diet, which may activate *Ifi27* transcription.

Genetic ablation of *Ifi27* in adipocytes caused a dramatic decrease in the number of mitochondrial cristae. The AKO mice housing at room temperature exhibited relatively normal metabolic parameters, indicating the adipocyte mitochondria with less cristae are still able to fulfill the cellular energy demand in the mild cold condition (22 °C). However, when challenged by severe cold (4 °C) exposure, loss of *Ifi27* ruined the metabolic plasticity in brown adipocytes, displaying dramatically decreased level and activity of the SDH complex, reduced ability to oxidize fatty acids, less oxygen consumption and compromised thermogenesis. All these data suggest when subjected to stress, the mitochondrial bioenergetic network in *Ifi27‐*knockout adipocytes cannot accommodate any more.

Maintenance of mitochondrial proteostasis is to monitor and control the mitochondrial protein biogenesis, import, folding and degradation, which is an indispensable mechanism of mitochondrial stress adaption.^[^ ^31^, ^32^ ^]^ Most of the mitochondrial proteins are translated on cytosolic ribosomes and imported as precursors into mitochondrion. To avoid aggregation or being insoluble, intramitochondrial molecular chaperones assist these nascent polypeptides folding or facilitate the degradation of misfolded or damaged proteins by proteases.^[^ ^33^ ^]^ The chaperone TRAP1 modulates mitochondrial energy metabolism and redox homeostasis that shields cells from oxidative stress.^[^ ^34^, ^35^, ^36^ ^]^ In cancer cells, deficiency of *Trap1* results in decreased SDHB level, increased production of ROS and rewriting cellular metabolism,^[^ ^20^, ^37^ ^]^ which is largely phenocopied by *Ifi27* deletion in brown adipocytes. The data here suggest IFI27 facilitates SDHB to interact with TRAP1 and thus stabilizes SDHB. IFI27 induction by cold exposure and HFD (Figure 1B,D) may represent an adaption to increased loss‐of‐proteostasis pressure, since SDHB is fragile to elevated ROS and inclined to conformational alteration due to the oxidation modifications.^[^ ^38^, ^39^ ^]^

Genetic *Sdh* gene mutation is tumorigenesis, and also implicated in neurodegeneration and diabetes.^[^ ^40^, ^41^, ^42^ ^]^ Tumor cells associated with *Sdh* mutations shut down the TCA cycle, but oxidize more glutamine or increase pyruvate carboxylase‐dependent pyruvate utilization to replenish TCA intermediates.^[^ ^43^, ^44^ ^]^ Compared to the SDH‐deficient cells, adipocytes with *Ifi27* deletion showed relatively minor metabolic remodeling possibly due to some remaining SDH activity. Increased succinate oxidation is a metabolic signature of adipocyte thermogenesis upon cold exposure, whereas inhibition of SDH suppresses UCP1‐dependent thermogenesis.^[^ ^45^ ^]^ Accumulated succinate inhibits lipolysis, induces protein succinylation or inflammatory.^[^ ^46^, ^47^ ^]^ It is reported that succinylated UCP1 impairs UCP1 activity and stability.^[^ ^48^ ^]^ Thus we reasoned that elevated levels of succinate may account for the compromised lipolysis and decreased UCP1 level in AKO mice BAT upon cold stress. The *Ifi27* knockout brown adipocytes also utilized less fatty acid to fuel thermogenesis as indicated by reduced levels of HSL phosphorylation and CPT1B, and less HADHA activity. Taken together, IFI27 integrates succinate and fatty acid oxidation to nourish thermogenesis.

One undetermined issue in this study is how ablation of *Ifi27* affects cristae structure. The alteration in the composition of PC and ether PE may convey a hint about the answer. PC and ether PE are synthesized in ER and peroxisome, and imported to destination organelles. PC (34:2) is the most plentiful PC in BAT mitochondria at room temperature, and it is further increased after mice are housing at 6.5 °C for 7 days.^[^ ^49^ ^]^ A reduction in mitochondrial PC or ether PE content is associated with abnormal mitochondrial function and morphogenesis.^[^ ^27^, ^50^ ^]^ Loss of *Ifi27* has a considerable impact on the glycerolphospholipid composition in BAT, indicating IFI27 deficiency may alter phospholipid synthesis pathway in ER and peroxisome. This is capable of happening since wide communication and cooperation among mitochondria, ER and peroxisome is observed via mitochondria‐associated membranes or mitochondrial‐derived vesicles.^[^ ^51^, ^52^, ^53^ ^]^ Investigation to define the mitochondrial lipid composition in *Ifi27*‐depleted adipocytes is worth carrying out in the future.

## 4 Conclusion

In summary, we have uncovered an essential role of IFI27 in controlling brown adipocyte mitochondrial metabolism and adaption to the changing environment. These findings broaden our horizons on IFI27 responsibility in thermogenic regulation and metabolic adaption in response to cold stimuli.

## 5 Experimental Section

### Animal Studies

All animal studies were performed in accordance with an Animal Care and Use Committee‐approved protocol of the Fudan University Shanghai Medical College. *Ifi27*‐floxed mice were prepared by Beijing Biocytogen Company on C57BL/6 background. To generate adipocyte‐specific *Ifi27* knockout mice, *Ifi27* ^flox/flox^ mice were crossed to mice expressing *Cre* recombinase under the control of the *Adiponectin* promoter (Jackson Laboratory, 028020). Mice were housed at 12‐h light/dark cycle (8:00 am to 8:00 pm lights on) at 22 °C with free access to water and a standard rodent chow diet. In each experiment, only the same gender and similar age littermate mice were used as control. For indirect calorimetry, *Ifi27* knockout mice and their littermate controls (WT) on a normal chow diet feeding were placed in the Comprehensive Lab Animal Monitoring System (Columbus Instruments) and allowed to acclimate for 24h. Then mice were monitored oxygen consumption, carbon dioxide production, and heat generation for 24h. Data were shown as the average over 12‐h dark and light cycle.

### APEX2 Electron Microscopy Imaging

The subcellular localization of IFI27–APEX2 was discerned by EM as previously described with minor modification.^[^ ^54^ ^]^ In brief, HEK293T cells that express IFI27–APEX2 were fixed by 2% glutaraldehyde for 60 min on ice and incubated in a Tris buffer containing diaminobenzidine (DAB, 1.4mm) and H_2_O_2_ (10mm) for 15–30 min, until light brown precipitates were shown in the cytoplasm under the light microscope. Then cells were rinsed five times with chilled buffer followed by staining with 1% osmium tetroxide for 30 min and 2% uranyl acetate overnight. The cell pellets were sent to the Wuhan Servicebio Company for embedding in resin and EM imaging. The TEM assay of adipose tissues was performed by the Electron Microscopy Core Laboratory in School of Basic Medical Science, Fudan University.

### Oxygen Consumption Assays In Vitro and Ex Vivo

Cultured brown adipocytes were trypsinized and pelleted by centrifugation at 500 g for 10 min. Freshly dissected brown adipose tissues were cut into small pieces or used for mitochondrion isolation. Cellular respiration was measured by suspending the cells or tissues in 1 mL phosphate‐buffered saline containing 25 mm glucose, 1 mm pyruvate, and 2% BSA with a Clark‐type oxygen electrode (Oxygraph^+^ system, Hansatech). Mitochondrial OCR on specific substrates were measured by incubating mitochondria with 10 mm succinate and 1 µm rotenone or 50 µm palmitoylcarnitine and 0.5 mm malate. The OCR was normalized by tissue weight, cell or mitochondrial protein content, respectively.

### Antibodies and shRNAs

Antibodies used in this study were listed in Table S3, Supporting Information. IFI27 polyclonal antibody was customized by Shanghai HuiOu Biotechnology Co. LTD. The polypeptide (amino acids 105 to 122) on IFI27 coupled to KLH was used as immunogen to subcutaneously inject New Zealand rabbits. The sera were collected after the fourth immunization, and the titer was determined by indirect ELISA. The shRNA targeting sequences were listed in Table S4, Supporting Information.

### Blue Native‐PAGE

Blue native‐PAGE was performed as described previously.^[^ ^55^ ^]^ Briefly, mitochondria were resuspended in lysis buffer (50 mm NaCl, 5 mm 6‐aminocaproic acid, 50 mm imidazole, 1 mm AEBSF, and protease inhibitor cocktail) with 2% digitonin. Lysates were resolved on a 4%–13% gradient native gel (Biorad, #4561083) using a running buffer without SDS. Proteins were transferred to PVDF membranes and blotted with the indicated antibodies.

### Mass Spectrometry (MS) Assay

BAT isolated from WT and AKO mice raised at ambient temperature were subject to lipidomic assay. BAT from 6 h cold‐exposed mice were subject to LC‐MS/MS assay to determine metabolite levels. Cell lysates of brown adipocytes expressing IFI27‐Flag were incubated with Flag beads at 4 °C for 4 h. The Flag beads were washed by buffer [200 mm NaCl, 0.5% Triton‐X‐100, 5% glycerol, 50 mm Tris‐HCl (pH 7.5), 1 mm PMSF] for four times. Then the attached proteins to Flag beads were identified by MS assay. All the MS assays were performed by Shanghai Applied Protein Technology Inc.

### SDH and HADHA Activity Assay

SDH activity was measured by a kit from Sango Biotech Inc. (D799376), and the assay was performed followed the manufacturer's instructions. The determination of HADHA activity was carried out according to the published method with slight modification.^[^ ^56^ ^]^ Briefly, the HADHA activity was evaluated as a measurement of decrease in the NADH. Freshly prepared mitochondria (30 µg mitochondria protein) or 0.5 µg purified HADHA‐GST protein was added to assay buffer [50 mm Tris‐HCl, 1 mM EDTA, 250 µµ NADH, 100 µm acetoacetyl CoA, 160 µµ DCIP (2,6‐dichlorophenolindophenol)]. After 3 min of incubation at room temperature, the change in NADH concentration was monitored by the decrease in absorbance at 600 nm due to DCIP reduction by NADH. Consumption of 1 nmol DCIP per minute per µg protein in the reaction system is defined as one unit of the HADHA activity.

### Adipocyte Culture and Differentiation

The immortalized brown preadipocytes were maintained in DMEM (Meilunbio Inc, MA0212‐2) with 10% fetal bovine serum (FBS). When adipogenic differentiation was required, preadipocytes were plated into culture dishes or plates at 70% confluency, following by feeding the differentiation medium the next day (DMEM supplemented with 10% FBS, 20 nm insulin, and 1 nm T3) for 2 days (day 2 to day 0). Then the Sinduction medium (differentiation medium plus 0.5 µm dexamethasone, 0.5 mm isobutylmethylxanthine, and 0.125 mm indomethacin) was applied to cells for another 2 days (day 0 to day 2). For the next 4 days (day 2 to day 6), cells were replenished to the differentiation medium every other day to get fully differentiated.

### ROS and Mitochondria Membrane Potential Analysis

ROS detection kit (Meilunbio Inc, MA0219) was used following the manufacturer's instructions. Briefly, brown adipocytes knocking down *Ifi27* by lentiviral transduction were incubated with 10 µm 2′‐7′dichlorofluorescin diacetate (DCFH‐DA) redox probe for 30 min at 37 °C. After three washes by serum‐free medium, cells were harvested and analyzed on fluorimeter at the specified wavelength. Mitochondria membrane potential was determined by JC1 (Meilunbio Inc, MA0338) staining. Mature adipocytes were washed by PBS and incubated in JC1‐containing medium for 30min at 37 °C. The mitochondria were illuminated by fluorescence microscopy at 488 nm and the emission was collected between 515–545 and 575–625 nm.

### Isolation of Mitochondria

Subcellular fractionation was achieved by following the manufacturer's instructions for the cell mitochondria isolation (Meilunbio Inc, MA0375). Briefly, adherent cells were collected by trypsin digestion and homogenized manually with dounce glass tissue grinders. After centrifugation of the homogenate at 700 g for 10 min, supernatant containing mitochondrial fraction was centrifuged at 10 000 g for 10 min at 4 °C to obtain the crude mitochondria precipitate. The precipitations were subjected to western blot analysis.

### Quantification of Mitochondrial Content, Diameter, and Cristae Abundance

For mitochondria content assay, genomic DNA was extracted from adipose tissues by DNA isolation kit (Vazyme Biotech Co., DC112). A certain amount of genomic DNA (about 100 ng) was used as templates for quantitative real‐time PCR to detect the mitochondria‐encoded gene *Cox2* and nuclear‐encoded gene *Cebpα* levels. The mitochondrial content is shown as the ratio of the mitochondrial DNA level relative to nuclear DNA level. For measuring mitochondrial diameter, Image J software was used to quantify the transverse diameter of each mitochondrion in the EM images. 60 mitochondria were measured in each group and the average diameter was shown. Cristae abundance was estimated by the ratio of inner to outer mitochondrial membrane perimeter.^[^ ^57^ ^]^ Thirty mitochondria were analyzed using Image J.

### Statistical Analysis

Statistical analyses were performed using GraphPad Prism software (v.8.0). Data are presented as mean ± standard error. Differences between two groups were assessed using unpaired two‐tailed Student's *t*‐test, one‐way or two‐way ANOVA as indicated. Statistical significance was shown as *p < 0.05, **p < 0.01, ***p < 0.001, or ****p < 0.0001.

## Conflict of Interest

The authors declare no conflict of interest.

## Author Contributions

X.C. performed most experiments, acquired and analyzed data. H.L., T.S., Q.Z., F.L., W.L., C.Y., H.H. and Q.T. aided in some experiments. D.P. designed the project, supervised the study and drafted the manuscript.

## Supporting information

Supporting Information

Supporting Information

## Data Availability

The data that support the findings of this study are available from the corresponding author upon reasonable request.
