# Supplementary material for: IFI27 Integrates Succinate and Fatty Acid Oxidation to Promote Adipocyte Thermogenic Adaption
Source: Adv Sci (Weinh). 2023 Aug 6;10(28):2301855. doi: 10.1002/advs.202301855 (PMC10558685; doi:10.1002/advs.202301855)
Supplement: Supplementary file 1 — Supporting Information [file ADVS-10-2301855-s001.pdf]

ADVANCED SCIENCE
Open Access

Supporting Information

for *Adv. Sci.*, DOI 10.1002/advs.202301855

IFI27 Integrates Succinate and Fatty Acid Oxidation to Promote Adipocyte Thermogenic Adaption

*Xuan Cui, Haojie Liu, Ting Shi, Qingwen Zhao, Feiyan Li, Wenjing Lv, Chao Yu, Haiyan Huang, Qi-Qun Tang and Dongning Pan**

Supporting Information

IFI27 integrates succinate and fatty acid oxidation to promote adipocyte thermogenic adaption

*Xuan Cui, Haojie Liu, Ting Shi, Qingwen Zhao, Feiyan Li, Wenjing Lv, Chao Yu, Haiyan Huang, Qi-Qun Tang, Dongning Pan**

The file includes:

Supplementary Figures and Figure Legends

Supplementary Tables

Fig. S1

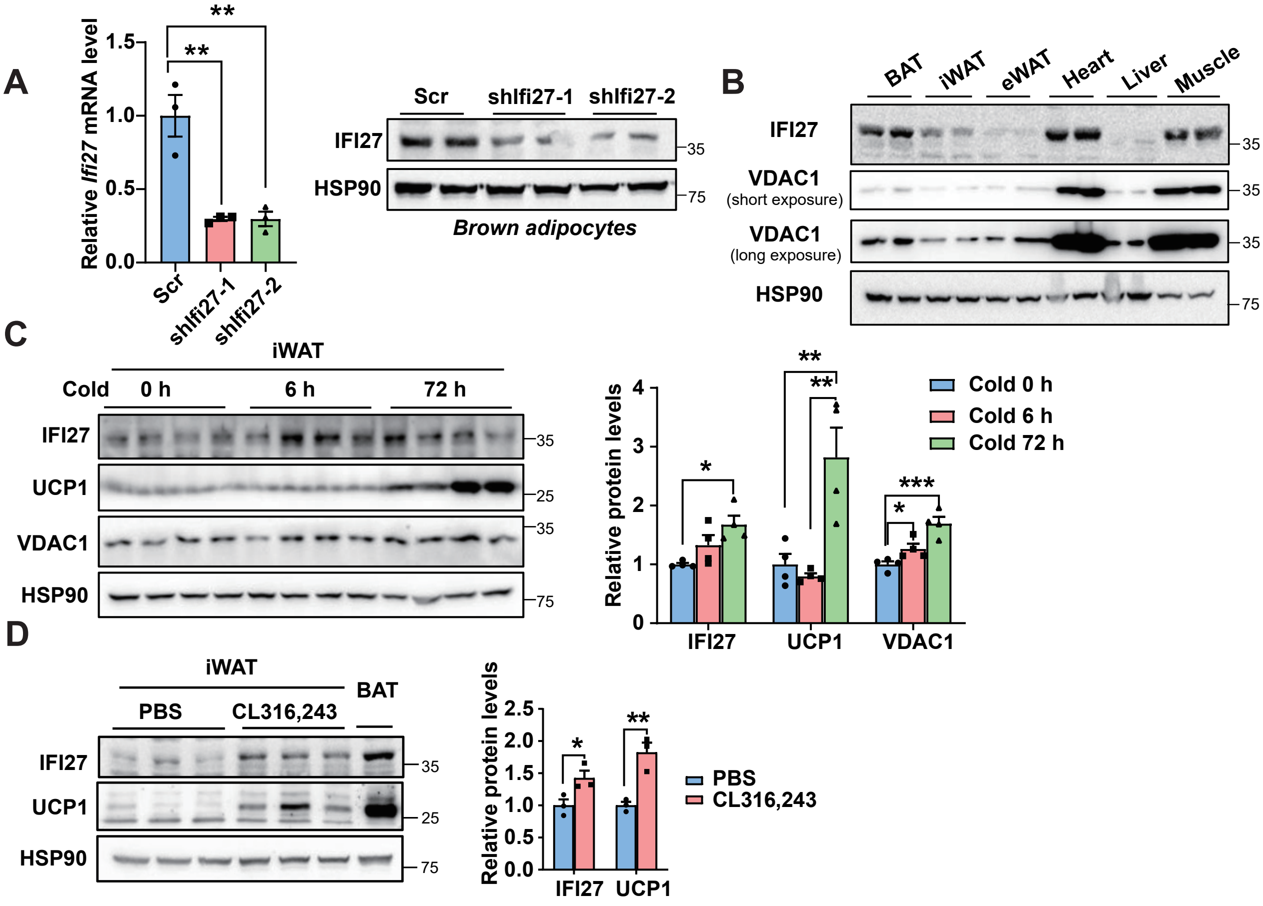

Figure S1. IFI27 is enriched in BAT and induced by β3-adrenergic signaling.

A. Lentivirus expressing sh*Ifi27* was used to transduce brown preadipocytes followed by induction to mature adipocytes. QPCR (n=3) and western blot analyzed the *Ifi27* level in the knockdown adipocytes.

B. Western blot analysis of the IFI27 and VDAC1 protein in mouse tissues. n=2, 8-week-old male mice.

C. IFI27, UCP1 and VDAC1 levels in iWAT after cold challenge for indicated time. Right: quantification of the protein levels. n=4 each group.

D. IFI27 and UCP1 protein levels in iWAT of 10-week-old male mice. BAT was used as a positive control. The mice were intraperitoneally injected Cl316,243 (0.5 mg kg^{-1} body weight) or phosphate-buffered saline (PBS) for 5 days. Right: quantification of IFI27 and UCP1 levels. n=3 each group.

For statistical analyses, one-way ANOVA analysis of variance and Tukey's post hoc tests were performed in (A, C), two-tailed unpaired Student's t test was performed in (D). The data shown are mean ± SEM. *$p < 0.05$; **$p < 0.01$; ***$p < 0.001$.

Fig. S2

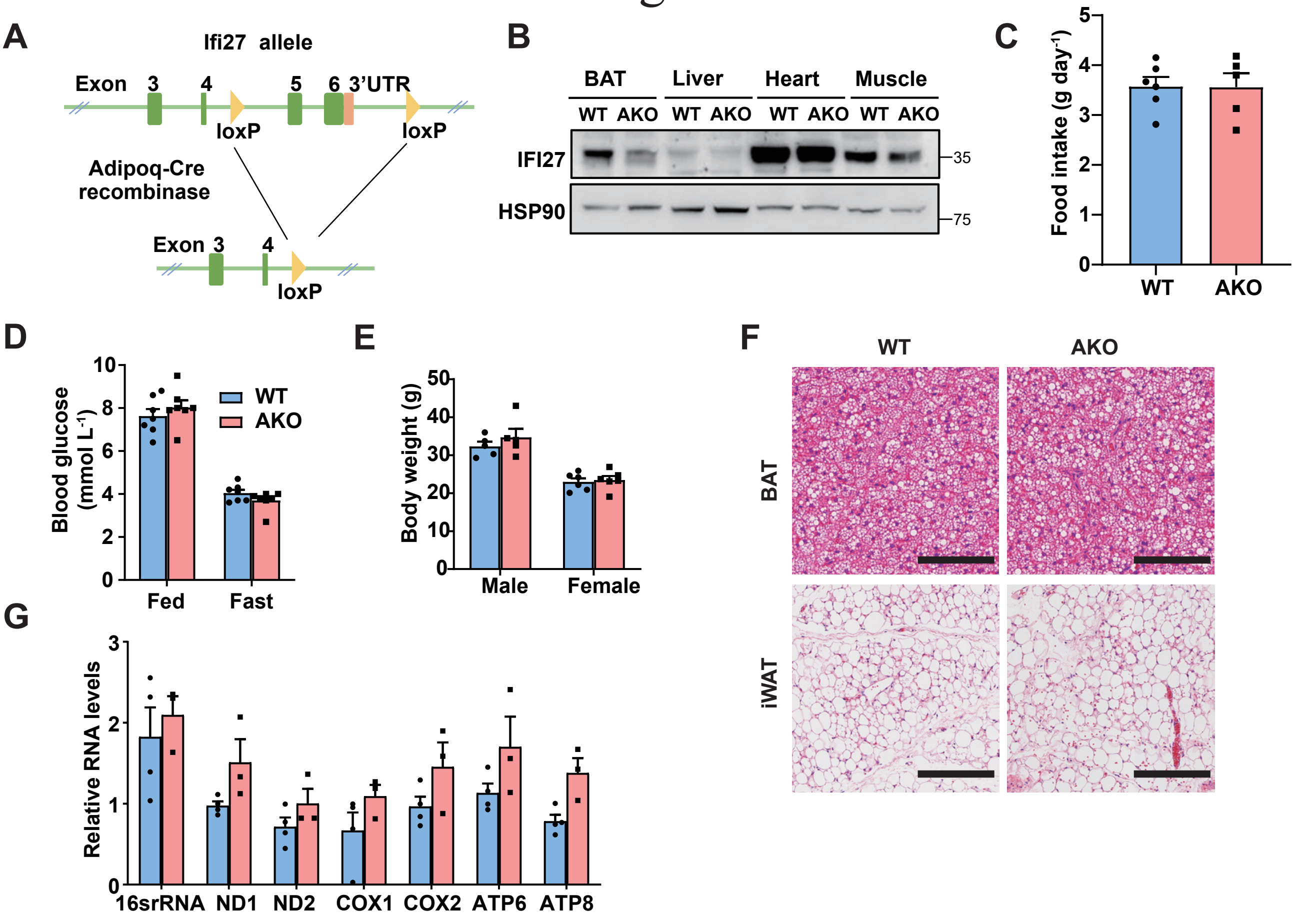
A
Ifi27 allele
Exon 3 4 5 6 3'UTR
loxP
loxP
Adipoq-Cre
recombinase
Exon 3 4
loxP
B
BAT Liver Heart Muscle
WT AKO WT AKO WT AKO WT AKO
IFI27
HSP90
35
75
C
Food intake (g day⁻¹)
WT AKO
D
Blood glucose (mmol L⁻¹)
WT
AKO
Fed Fast
E
Body weight (g)
Male Female
F
WT AKO
BAT
iWAT
G
Relative RNA levels
16srRNA ND1 ND2 COX1 COX2 ATP6 ATP8

Figure S2. Adipocyte-specific *Ifi27* knockout mice show relatively normal metabolic phenotype at room temperature.

A. Genetically inactivation of *Ifi27* in adipocytes was achieved by breeding the $Ifi27^{flox/flox}$ mice to animals expressing *Adipoq-Cre* recombinase. Littermates without *Cre* expression ($Ifi27^{flox/flox}$) were used as controls.

B. Western blot revealed the IFI27 level in tissues of WT and AKO mice.

C,D. Food intake (C) and blood glucose level (D) in 8-week-old male WT and AKO mice. n=5-7 each genotype.

E. Body weight of 24-week-old male and female WT and AKO mice housed at room temperature. n=5-6 each genotype.

F. Haemotoxylin and eosin staining of BAT and iWAT from 11-week-old male mice. Scale bar: BAT 100 μm; iWAT 200 μm.

G. Relative mRNA levels of mitochondrial DNA-encoding genes in BAT. n=3-4 each genotype.

For statistical analyses, two-tailed unpaired Student's t test was performed. The data shown are mean ± SEM.

Fig. S3

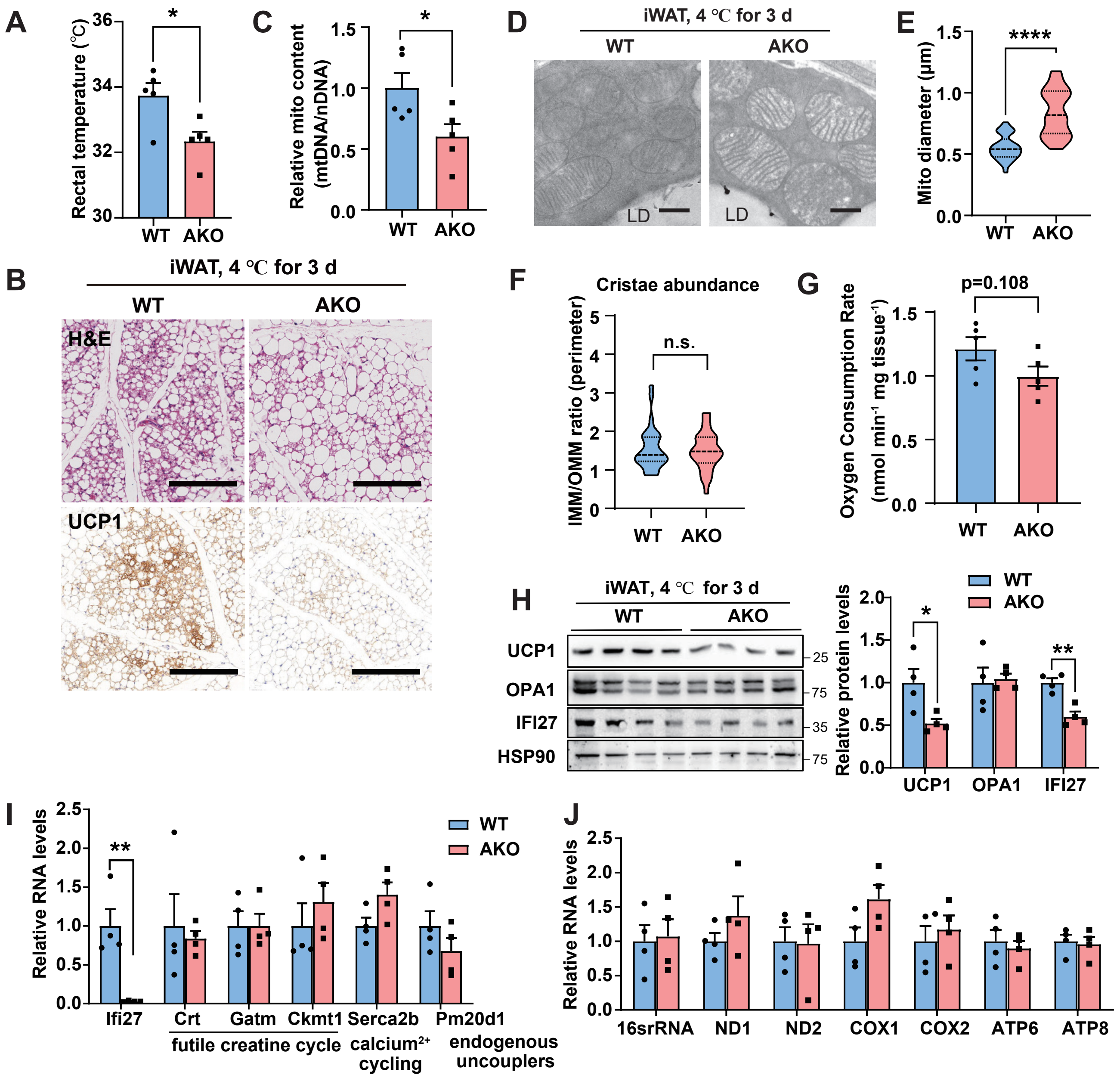
A
Rectal temperature (℃)
WT
AKO
C
Relative mito content (mtDNA/nDNA)
D
iWAT, 4 ℃ for 3 d
LD
E
Mito diameter (μm)
****
B
H&E
UCP1
F
Cristae abundance
IMM/OMM ratio (perimeter)
n.s.
G
p=0.108
Oxygen Consumption Rate
(nmol min-1 mg tissue-1)
H
OPA1
IFI27
HSP90
Relative protein levels
I
Relative RNA levels
Ifi27
Crt
Gatm
Ckmt1
Serca2b
Pm20d1
futile creatine cycle
calcium2+ cycling
endogenous uncouplers
J
16srRNA
ND1
ND2
COX1
COX2
ATP6
ATP8

Figure S3. WAT beiging in *Ifi27*-AKO mice is impaired.

A. Rectal temperature of 8-week-old male WT and AKO mice at 4°C for 3 days. n=5 each genotype.

B. Haemotoxylin and eosin staining and UCP1 immunostaining of iWAT from WT and AKO mice following 3 days cold exposure. Scale bar: 200 μm.

C. Mitochondrial content in iWAT of mice in (A). n=5 each genotype.

D-F. Representative EM images showed the mitochondrial morphology in iWAT (D). Mitochondrial diameter (E, n=30) and cristae abundance (F, n=30) were quantified by Image J. The mice were exposed to 4ºC for 3 days. LD: lipid droplet. Scale bar: 500 nm. n.s.: not significant.

G. Mice were treated as in (A). iWAT oxygen consumption rate was measured by Clark-type oxygen electrodes. n=4 each genotype.

H. Mice were treated as in (A). Western blot analysis detected levels of the indicated proteins in iWAT. The right panel showed the protein quantification. n=4 each genotype.

I. Quantitative RT-PCR screening of genes involved in Ucp1-independent thermogenic program in iWAT of WT and AKO mice following 3 days cold exposure. n=4 each genotype.

J. Relative mRNA levels of genes encoded by mtDNA in iWAT of WT and AKO mice following 3 days cold exposure. n=4 each genotype.

For statistical analyses, two-tailed unpaired Student's t test was performed. The data shown are mean ± SEM. $^*p < 0.05$; $^{**}p < 0.01$; $^{****}p < 0.0001$.

Fig. S4

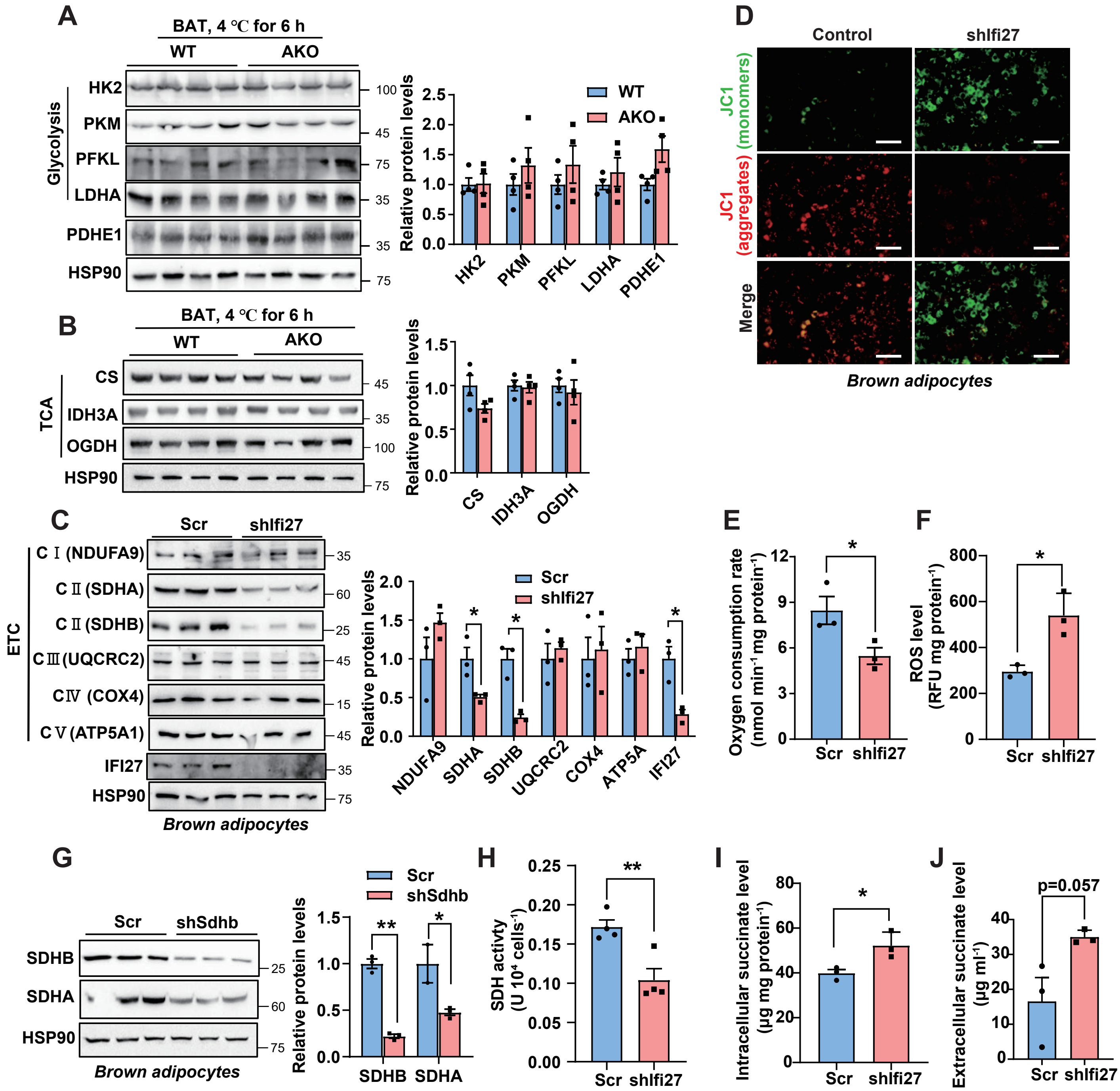

A
BAT, 4 ℃ for 6 h
WT
AKO
Glycolysis
HK2
PKM
PFKL
LDHA
PDHE1
HSP90
Relative protein levels
WT
AKO
B
TCA
CS
IDH3A
OGDH
C
Scr
shIfi27
ETC
C Ⅰ (NDUFA9)
C Ⅱ (SDHA)
C Ⅱ (SDHB)
C Ⅲ (UQCRC2)
C Ⅳ (COX4)
C Ⅴ (ATP5A1)
IFI27
Brown adipocytes
NDUFA9
SDHA
SDHB
UQCRC2
COX4
ATP5A
D
Control
JC1 (monomers)
JC1 (aggregates)
Merge
E
Oxygen consumption rate (nmol min⁻¹ mg protein⁻¹)
F
ROS level (RFU mg protein⁻¹)
G
shSdhb
H
SDH activty (U 10⁴ cells⁻¹)
I
Intracellular succinate level (μg mg protein⁻¹)
J
Extracellular succinate level (μg ml⁻¹)
p=0.057

Figure S4. Knockdown of *Ifi27* decreases the SDHB level *in vitro*.

A,B. Western blot analysis of indicated proteins and their quantification in BAT of mice after 6 h cold exposure. n=4 each genotype.

C. Western blot analysis of the representative ETC subunits in brown adipocytes (left). Immortalized brown preadipocytes were knocked down *Ifi27* expression by lentiviral shRNAs. Then cells were induced to mature adipocytes following by western blot analysis on day 6. The right panel showed the protein quantification. n=3 each group.

D. The mitochondrial membrane potential analysis by JC1 staining in *Ifi27*-knockdown brown adipocytes. The knockdown cells were generated as in (C).

E,F. Oxygen consumption rate (E) and ROS level (F) in *Ifi27*-knockdown adipocytes. n=3 each group.

G. Lentiviral shRNAs against *Sdhb* infected preadipocytes. SDHA and SDHB levels were detected (left) and quantified (right) in mature adipocytes on differentiation day 6. n=3 each group.

H-J. SDH activity (H), intracellular (I) and extracellular succinate levels (J) were measured in *Ifi27*-knockdown brown adipocytes. n=3-4 each genotype.

For statistical analyses, unpaired two-tailed Student's t tests were performed. The data shown are mean ± SEM. *$p < 0.05$; **$p < 0.01$.

Fig. S5

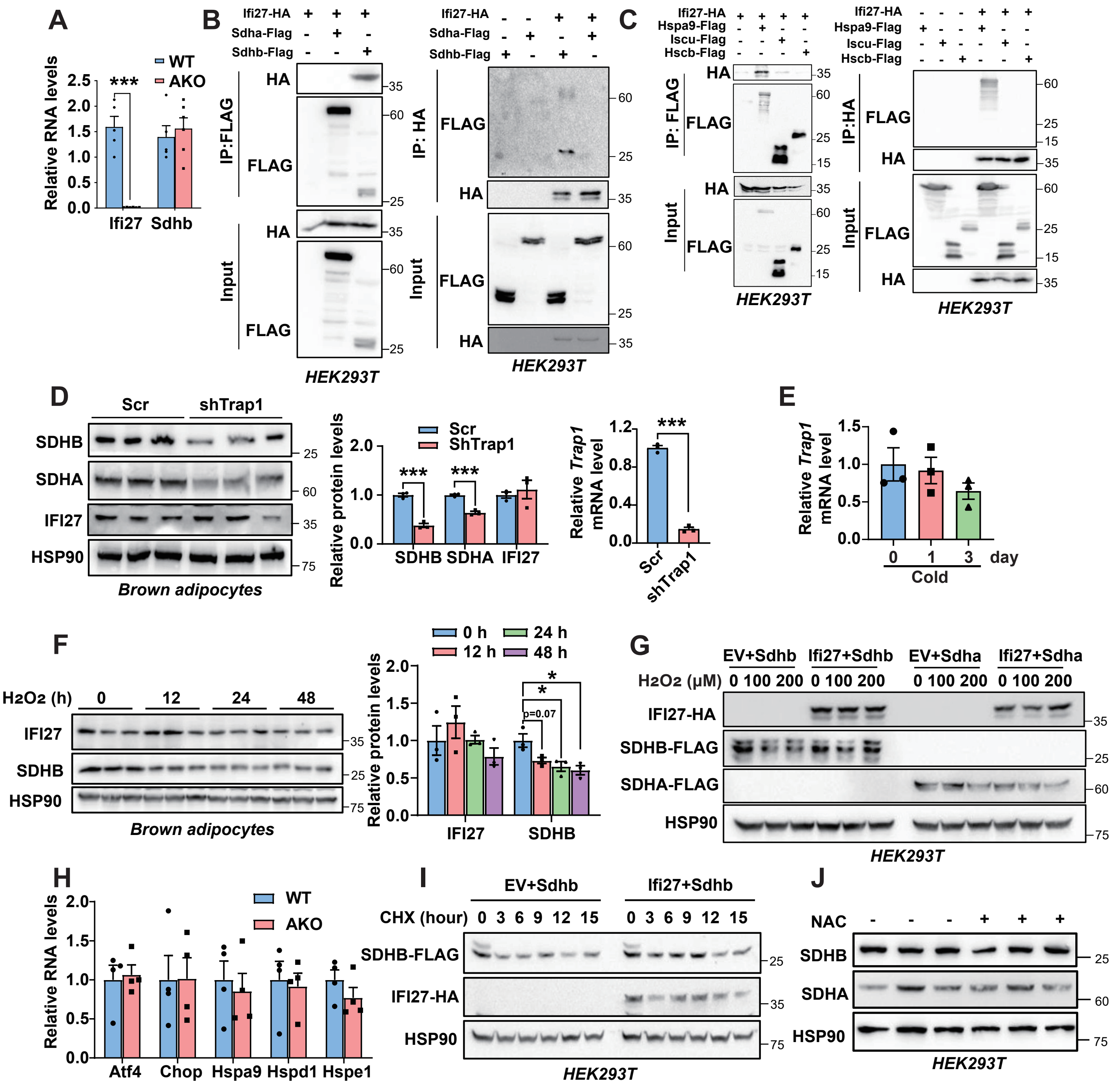
A
Relative RNA levels
WT
AKO
***
Ifi27 Sdhb
B
Ifi27-HA
Sdha-Flag
Sdhb-Flag
IP:FLAG
Input
IP: HA
HA
FLAG
HEK293T
C
Hspa9-Flag
Iscu-Flag
Hscb-Flag
IP: FLAG
IP:HA
D
Scr
shTrap1
SDHB
SDHA
IFI27
HSP90
Brown adipocytes
Relative protein levels
ShTrap1
Relative Trap1 mRNA level
E
day
Cold
F
H2O2 (h)
0 h
12 h
24 h
48 h
p=0.07
G
EV+Sdhb
Ifi27+Sdhb
EV+Sdha
Ifi27+Sdha
H2O2 (μM)
IFI27-HA
SDHB-FLAG
SDHA-FLAG
H
Atf4
Chop
Hspa9
Hspd1
Hspe1
I
CHX (hour)
J
NAC

Figure S5. IFI27 stabilizes SDHB in face of stress

A. Relative mRNA levels of *Ifi27* and *Sdhb* in BAT of WT and AKO mice. The mice were exposed to 4ºC for 6 h without food. n=5-6 each genotype.

B,C. Plasmids were transfected into HEK293T cells. The interaction between IFI27 and indicated proteins was analyzed by immunoprecipitation assay that was performed 48 h after transfection.

D. Western blot analysis (left) of indicated proteins and their quantification (middle) in immortalized brown adipocytes expressing lentiviral shRNAs targeting *Trap1*. Right panel: the relative mRNA level of *Trap1* in *Trap1*-knockdown mature brown adipocytes. n=3 each group.

E. Relative *Trap1* mRNA level in BAT of the mice which were exposed to cold for 0 to 3 days. n=3 each group.

F. Hydrogen peroxide (H_2O_2 100 μM) treated the brown adipocytes for indicated time before the western blot analysis.

G. The indicated plasmids were transfected into HEK293T cells. H_2O_2 treated the cells for 12 h before the western blot analysis.

H. Relative mRNA levels of the indicated genes in BAT of WT and AKO mice. The mice were exposed to 4ºC for 6h. n=4 each genotype.

I. SDHB and IFI27 were ectopically expressed in HEK293T cells by plasmid transfection. Cycloheximide (CHX) treated cells for indicated time followed by western blot analysis.

J. Brown preadipocytes were induced to differentiation by standard protocol with or without supplementing N-acetyl cysteine (NAC, 2mM). SDHA and SDHB protein levels were analyzed by western blot on day 6.

For statistical analyses, two-tailed unpaired Student's t test was performed in (A, D). One-way ANOVA analysis of variance and Tukey's post hoc tests were performed in (F), The data shown are mean ± SEM. *$p < 0.05$; ***$p < 0.001$.

Fig. S6

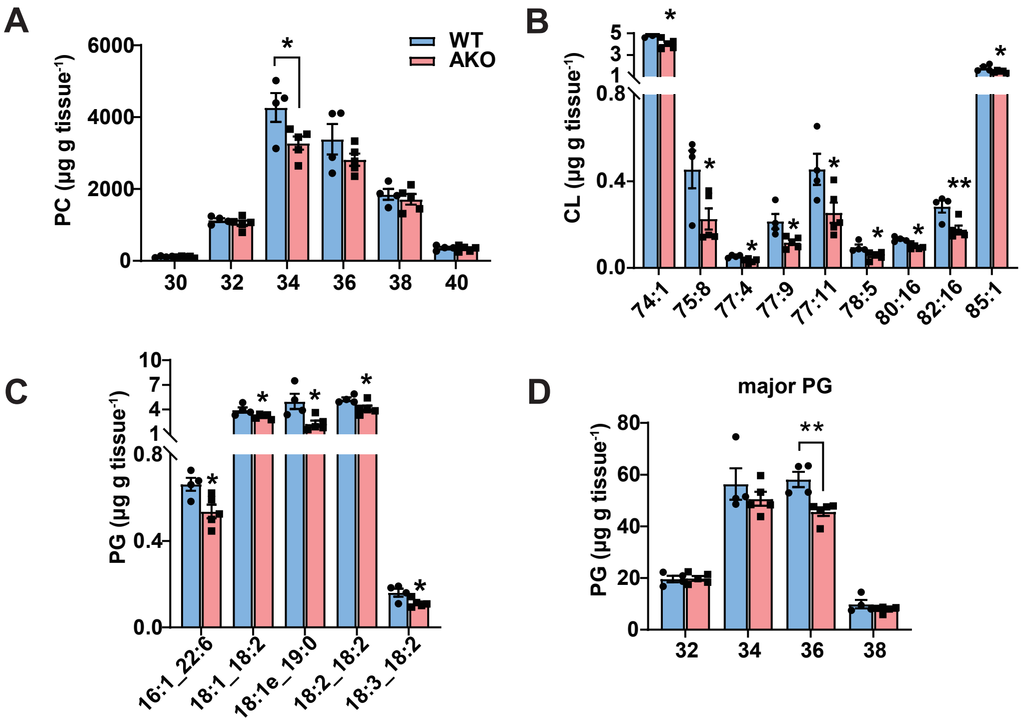

Figure S6. IFI27 ablation changes the composition of lipid species in BAT

A. The abundance of major PC species in BAT of WT (n=4) and AKO (n=5) mice.

B. The abundance of the indicated CL species in BAT of WT (n=4) and AKO (n=5) mice.

C,D The abundance of the indicated PG species in BAT of WT (n=4) and AKO (n=5) mice.

For statistical analyses, two-tailed unpaired Student's t test was performed. The data shown are mean ± SEM. *p < 0.05; **p < 0.01.

Table S1. IFI27 interacting proteins identified by IP-MS

	Accession	Gene Name	Coverage [%]	# Peptides	# PSMs	# Unique Peptides	Abundances (Grouped): IFI27-1	Abundances (Grouped): IFI27-2	Abundances (Grouped): Control
1	P38647	Hspa9	58	38	187	38	1037070233.9	995372055.6	756599431.7
2	Q8BMS1	Hadha	56	27	84	27	250030303.2	217566192.4	164005674.6
3	P63038	Hspd1	47	20	48	20	71872369.2	78754832.1	55709347.2
4	D3Z041	Acsl1	36	20	42	20	45068662.9	47129897.9	35098094.0
5	Q8BVI9	Slc25a4	34	8	21	2	2863431.2	2656029.0	1933125.2
6	E9Q1C5	Ifi27	32	5	15	5	373630645.3	358777861.0	2436438.5
7	P51175	Ppox	24	7	10	7	4888325.5	11038065.6	4055904.5
8	Q8BWM0	Ptges2	23	6	11	6	9898547.0	8678278.2	6712477.3
9	P18155	Mthfd2	19	5	10	5	59369880.3	59155660.5	47178235.5
10	G5E895	Akr1b10	16	5	11	2	22017551.0	21201729.6	13820776.3
11	O70325	Gpx4	13	2	3	2	4105422.6	4143945.6	2923584.4
12	B1AU25	Aifm1	12	5	7	5	4417812.4	4453928.8	2958593.6
13	Q9CQA3	Sdhb	12	3	7	3	4848256.3	3993942.3	3257724.9
14	Q80ZS3	Mrps26	10	2	3	2	726357.2	661384.6	484515.3
15	A0A0J9YV64	Polg	10	1	1	1	73902723.9	62241656.0	50059212.2
16	Q3ULD5	Mccc2	7	3	5	3	1245876.8	1332441.3	720431.1
17	G3X9M0	Dap3	7	2	4	2	1179790.7	717457.4	286735.8
18	Q78PY7	Snd1	2	2	2	2	888143.5	758497.2	567787.5
19	Q64521	Gpd2	2	2	5	2	1906992.7	1694166.3	1398418.1
20	Q3U2U4	Mrps5	2	1	1	1	974418.3	725779.1	566534.0
21	P54071	Idh2	2	1	2	1	438103.4	473444.7	358295.0

Table S3 Antibodies used in this study

Company	Antibody	Product code
Abcam	anti-UCP1	ab10983
Cell Signaling Technology	anti-p-HSL(Ser660)	#45804
Proteintech	anti-VDAC1	55259-1-AP
	anti-HSP90	13171-1-AP
	anti-α-TUBULIN	66031-1-Ig
	anti-OGDH	15212-1-AP
	anti-CS	16131-1-AP
	anti-IDH3A	15909-1-AP
	anti-NDUFA9	20312-1-AP
	anti-SDHA	14865-1-AP
	anti-SDHB	10620-1-AP
	anti-UQCRC2	14742-1-AP
	anti-COX7A1	11413-1-AP
	anti-ATP5A	66037-1-Ig
	anti-CHCHD10	25671-1-AP
	anti-HK2	22029-1-AP
	anti-PKM	15821-1-AP
	anti-PFKL	15652-1-AP
	anti-LDHA	19887-1-AP
	anti-PDHA1	18068-1-AP
	anti-HSL	17333-1-AP
	anti-ATGL	55190-1-AP
	anti-CPT1B	22170-1-AP
	anti-HSPA9	14887-1-AP
	anti-FLAG	20543-1-AP
	anti-GST	10000-0-AP
	anti-LONP1	15440-1-AP
	anti-HADHA	10758-1-AP

Table S4. Small hairpin RNA targeting sequence used in this study

**shRNA**	**target sequence**
shIfi27-1	CCTCTCTAGCAGCTAAGATGA
shIfi27-2	TCTAACACAGAGAAGGATTAA
shSdhb	CCCTGATTTGAGTAACTTCTA
shLonp1	CCTGAGAACCTACAAGACTTT
shTrap1	GCTGACAAGGTTGAAGTCTAT
